# Supplementary material for: Skmer: assembly-free and alignment-free sample identification using genome skims
Source: Genome Biol. 2019 Feb 13;20:34. doi: 10.1186/s13059-019-1632-4 (PMC6374904; doi:10.1186/s13059-019-1632-4)
Supplement: Supplementary file 1 — Supplementary material (PDF 1209 kb) [file 13059_2019_1632_MOESM1_ESM.pdf]

Supplementary Material

Appendix A: Theoretical results

Consider two genomes of identical length L and separated by hamming distance D where the hamming distance is defined as the fraction of variant sites between the perfect alignment of the two genomes. We would like to estimate D from two genome-skims.

Mutations

We model the two genomes as the outcome of a random process that copies a genome and introduces mutations at each position i.i.d with a fixed probability d. Indexing from left to right, we can define $n = L - k + 1$ k-mers (note that $n \approx L$ for any reasonable choice of k and genome length). Let X_i be a binary random variable (r.v.) that indicates whether k-mer i is identical between the two genomes. Clearly, in our model, $X_i \sim \text{Bern}(p)$ where $p = (1-d)^k$. Then, $W = \sum_1^n X_i$ gives the number of shared k-mers. If J is defined as the Jaccard index over the set of all k-mers from both genomes, it's easy to see that $J = \frac{W}{2n-W}$ and thus, $\frac{W}{n} = \frac{2J}{1+J}$. We further make a simplifying assumption. We assume all X_i r.v.s are independent, an assumption that is true for most pairs of k-mers but ignores the fact that each k-mer overlaps with k-1 other k-mers. With this assumption, the maximum likelihood estimate of p is simply

$$\hat{p} = \frac{W}{n} = \frac{2J}{1+J} \ .$$

By the functional invariance of maximum likelihood, the ML estimate of d is given by:

$$\hat{d} = 1 - \left(\frac{2J}{1+J}\right)^{\frac{1}{k}} \ .$$

k-mer sampling

We now assume that each genome is covered uniformly at random. Thus, k-mers are also sub-sampled and we assume each k-mer is sampled at least once with probability η_1 in the first genome and η_2 in the second genome; we derive the relationship between these probabilities and genome coverage below. We estimate

η values separately (also described below) and here consider them as given. For each $1 \leq i \leq n$ and $j \in \{1, 2\}$, let $Y_{j,i} \sim \text{Bern}(\eta_j)$ be the indicator of whether the k-mer i is sampled at least once in the genome j. Under this scenario, the number of k-mers shared between the two genomes is given by the r.v. $W = \sum_1^n X_i Y_{1,i} Y_{2,i}$. Defining $Z = X_i Y_{1,i} Y_{2,i}$, we get $W = \sum_1^n Z_i$ and $Z_i \sim \text{Bern}(r)$ where $r = p\eta_1\eta_2$ by the independence of the mutation process and each of the two k-mer sampling processes. Assuming independence between Z_i r.v.s (again ignoring the overlap between consecutive k-mers) we get the ML estimate $\hat{r} = \frac{W}{n}$, and thus (for a given η_1 and η_2) we have

$$\hat{r} = \hat{p}\eta_1\eta_2 = \frac{W}{n} \tag{S1}$$

Let $U = \sum_1^n S_i$ where $S_i = Y_{1,i} + Y_{2,i} - Y_{1,i} Y_{2,i} X_i$. It is easy to see that U gives the total number of sampled k-mers in both genomes. However, S_i is not a Bernoulli and thus, U is not Binomial. Nevertheless, the same assumptions that we used to treat X_i and Z_i r.v.s as independent also give us independence between S_i values; therefore, by the central limit theorem, $\frac{U}{n}$ can be approximated by a Gaussian with mean $q = \mathbb{E}[S_i]$. Moreover, $\mathbb{E}[S_i] = \mathbb{E}[Y_{1,i}] + \mathbb{E}[Y_{2,i}] - \mathbb{E}[Y_{1,i} Y_{2,i} X_i] = \eta_1 + \eta_2 - \eta_1\eta_2 p$ (note that X_i, $Y_{1,i}$ and $Y_{2,i}$ are independent). By this Gaussian approximation, the ML estimate of q given η_1, η_2 is given by:

$$\hat{q} = \eta_1 + \eta_2 - \eta_1\eta_2\hat{p} = \frac{U}{n} \ . \tag{S2}$$

Note that $J = \frac{W}{U}$. Equations S1 and S2 give two different ML estimators of the same parameter p given two different types of data (W and U). While the two estimators are not the same, because n is extremely large, both estimators have a very low variance. Exploiting the low variance, we treat the two estimates of p as equal and divide both sides of Equation S1 by Equation S2 to get:

$$\frac{\hat{r}}{\hat{q}} = \frac{W}{U} = J = \frac{\hat{p}\eta_1\eta_2}{\eta_1 + \eta_2 - \eta_1\eta_2\hat{p}} \ .$$

Solving for $\hat{p}$ and replacing $\hat{d} = 1 - \hat{p}^{\frac{1}{k}}$ gives

$$\hat{d} = 1 - \left(\frac{(\eta_1 + \eta_2)J}{\eta_1\eta_2(1 + J)}\right)^{\frac{1}{k}} .$$

Note that we have assumed a known coverage and thus we are not co-estimating η_j's and d. In practice, we need to first estimate η_1 and η_2, and we do it as we will describe.

Connection of η to read coverage

A k-mer stretching from position y to $y + k$ on the genome is covered by the reads that start in the interval $[y + k - \ell, y]$. Assuming that there is no sequencing error, and a uniform spread of of the N reads across the genome of length L. We show that the probability η that a k-mer is sampled by at least one read is given by

$$\eta = 1 - e^{-c(1-\frac{k}{\ell})}$$

Let X be a r.v. denoting the number of reads that cover a specific k-mer. Assuming a uniform spread of N reads across the genome of length L, the probability of x reads covering a k-mer (starting in an interval of length $\ell - k$) is given by

$$Prob(X = x) = \binom{N}{x}\left(\frac{l-k}{L}\right)^x\left(1 - \frac{l-k}{L}\right)^{N-x}$$

As N is large and $\frac{N(l-k)}{L}$ is constant, it can be closely approximated by

$$Prob(X = x) = \frac{\lambda^x}{x!}e^{-\lambda}$$

where $\lambda = \frac{N(l-k)}{L}$ is the k-*mer* coverage, and is related to the coverage c by

$$\lambda = \frac{l-k}{l}c$$

As the number of reads covering a k-mer follows Poisson distribution, the fraction of k-mers covered by 1 or more reads is

$$\eta = 1 - e^{-\lambda} \tag{S3}$$

Sequencing error

We model the sequencing error as an i.i.d process that corrupts each position of each read with a fixed probability ϵ. To extend our previous results to cover this

scenario, we need to see how the intersection r.v. (W) and the union r.v. (U) get affected.

We start with the intersection (W). We change the meaning of η to denote the probability that a k-mer is covered by at least one error-free read. The probability of a k-mer within a read being error-free is clearly

$$\rho = (1-\epsilon)^k \simeq e^{-k\epsilon} \tag{S4}$$

By conditioning on the number of reads covering a k-mer, the probability of not covering a k-mer with an error-free read is given by

$$\begin{aligned} Prob(\text{no error-free read}) &= \sum_{i=0}^{\infty} Prob(\text{all reads have error}|i \text{ reads})\, Prob(i \text{ reads}) \\ &= \sum_{i=0}^{\infty} (1-\rho)^i\, Prob(i \text{ reads}) \\ &= \sum_{i=0}^{\infty} (1-\rho)^i \frac{\lambda^i}{i!} e^{-\lambda} \\ &= e^{-\lambda\rho} \end{aligned} \tag{S5}$$

Hence, the probability that a k-mer is covered by at least one error-free read is given by

$$\eta = 1 - e^{-\lambda\rho} \tag{S6}$$

Note that Eqn. S6 reduces to Eqn. S3 when there is no sequencing error, i.e., $\rho = 1$. Similar to the case of no error, given η_1 and η_2, the r.v. $\frac{W}{n}$ (where W is the number of shared k-mers) can be used with Equation S1 to estimate r.

We now turn to the union (r.v. U). For large enough k, and for genomes that are random and repeat-free, with high probability ($> 1 - \frac{2L}{4^k}$) an error produces a new k-mer that is not observed in either of the input genomes. Ignoring the exceedingly unlikely event that two errors produce the same k-mer or that they produce a k-mer present in one of the two genomes, we can assume that the sequencing error generates as many new k-mers as the number of reads being affected by errors.

In the regime that includes errors, $U = \sum_1^n (T_{1,i} + T_{2,i}) - W$ where the r.v.s $T_{1,i}$ and $T_{2,i}$ give the total number of k-mers generated from the position i from the

first and second genomes, respectively. W.l.o.g, consider $T_{1,i}$. By conditioning on the number of reads covering a k-mer we have

$$\mathbb{E}[T_{1,i}] = \mathbb{E}[\mathbb{E}[T_{1,i}|x \text{ reads}]] = \sum_{x=0}^{\infty} \mathbb{E}[T_{1,i}|x \text{ reads}]\, Prob(x \text{ reads}) \tag{S7}$$

Given that x reads are covering a k-mer, $T_{1,i}$ equals the number of erroneous k-mers E, plus 1 if there is any error-free k-mer. As $E \sim Binom(x, 1-\rho)$

$$\begin{aligned}\mathbb{E}[T_{1,i}|x \text{ reads}] &= \sum_{j=0}^{x} (j + \mathbf{1}_{j \neq x}) \binom{x}{j} (1-\rho)^j \rho^{x-j} \\ &= x(1-\rho) + (1 - (1-\rho)^x)\end{aligned} \tag{S8}$$

and substituting into (S7)

$$\begin{aligned}\mathbb{E}[T_{1,i}] &= \sum_{x=0}^{\infty} ((1 - (1-\rho)^x) + x(1-\rho))\, Prob(x \text{ reads}) \\ &= \sum_{x=0}^{\infty} ((1 - (1-\rho)^x) + x(1-\rho)) \frac{\lambda_1^x}{x!} e^{-\lambda_1} \\ &= 1 - e^{-\lambda_1 \rho} + \lambda_1(1-\rho) \\ &= \eta_1 + \lambda_1(1-\rho) \\ &= \eta_1 + \lambda_1(1 - (1-\epsilon)^k)\end{aligned} \tag{S9}$$

Letting $\zeta_1 = \mathbb{E}[T_{1,i}]$ and using the same central limit argument we used before, $\frac{U}{n}$ becomes approximately a Gaussian with expectation $\zeta_1 + \zeta_2 - \eta_1\eta_2 p$. Similar to Equation S2, given ζ_1, ζ_2, η_1, and η_2, the Gaussian approximation gives us:

$$\zeta_1 + \zeta_2 - \eta_1\eta_2\hat{p} = \frac{U}{n}\ . \tag{S10}$$

Again, assuming that estimates of p in Equation S1 (with the new definition of η) and Equation S10 are the same (due to low variance), we divide the two equations and solve for d to get the estimator:

$$D = 1 - \left(\frac{(\zeta_1 + \zeta_2)J}{\eta_1\eta_2(1+J)} \right)^{1/k}\ .$$

Excluding low-copy k-mers from the Jaccard index calculation

If we discard k-mers observed less than m times, then a k-mer will survive if it is covered by m or more error-free reads. Hence, η becomes the probability of m or

more error-free reads covering a k-mer

$$\begin{aligned}\eta &= 1-\sum_{t=0}^{m-1} Prob(t \text{ error-free read})\\ &= 1-\sum_{t=0}^{m-1}\sum_{i=t}^{\infty} Prob(t \text{ error-free read}|i \text{ reads})\, Prob(i \text{ reads})\\ &= 1-\sum_{t=0}^{m-1}\sum_{i=t}^{\infty}\binom{i}{t}p^t(1-p)^{i-t}\frac{\lambda^i}{i!}e^{-\lambda}\\ &= 1-\sum_{t=0}^{m-1}\frac{(\lambda p)^t}{t!}e^{-\lambda p}\end{aligned} \tag{S11}$$

In general, we have shown that the probability distribution of the number of error-free k-mers is a Poisson with parameter λp.

Appendix B: Computing GTR distances

To compute the GTR matrix using the log-det approach, we need a 4×4 matrix F where each element is the fraction of sites where one genome has one letter while the other genome has the other letter. Given this matrix, $d = -\log(\det(F))$.

As elsewhere, we assume a no-indel scenario so that each k-mer mismatch can be attributed to a single nucleotide substitution. For $i, j \in \{\text{A,C,G,T}\}$, let $x_{ij} = x_{ji}$ denote the number of mutations of the form $i \leftrightarrow j$. Our goal is to estimate x_{ij} for all i, j. However, the paradigm of computing distance by hashing/sketching k-mers treats all mutations alike. Formally, the estimated distance d equals

$$d = x_{\text{AC}} + x_{\text{AG}} + x_{\text{AT}} + x_{\text{CG}} + x_{\text{CT}} + x_{\text{GT}}$$

We do the following:

1 Replace G and T with C, and compute distance $d_{\text{A}} = x_{\text{AC}} + x_{\text{AG}} + x_{\text{AT}}$.

2 Replace G and T with A, and compute distance $d_{\text{C}} = x_{\text{AC}} + x_{\text{CG}} + x_{\text{CT}}$.

3 Replace G with T, and compute distance $d_{\text{AC}} = x_{\text{AC}} + x_{\text{AG}} + x_{\text{AT}} + x_{\text{CG}} + x_{\text{CT}}$.

Combining, we get

$$x_{\text{AC}} = d_{\text{A}} + d_{\text{C}} - d_{\text{AC}}$$

A similar procedure can be used to compute all x_{ij} and normalization gives us F.

Note that this procedure reduces the space of possible k-mers of length k to 2^k possibilities instead of 4^k. Therefore, it will likely be required that k is increased for high accuracy when this approach is used.

Appendix C: Supplementary method details and commands

Here we provide the exact procedures and commands that we used to run external softwares throughout our experiments.

Simulating genome-skims using ART

To simulate short reads with length $\ell = 100$ and (default) error profiles of Illumina HiSeq2000, we ran

```
art_illumina -i FASTA_FILE -l 100 -f c -o FASTQ_FILE
```

To simulate reads with constant error rate $\epsilon = 0.01$ (Phred score = 20) at coverage c, we used

```
art_illumina -i FASTA_FILE -l 100 -qL 20 -qU 20 -f c -o FASTQ_FILE
```

Computing k-mer frequencies using JellyFish

To count all k-mers of length $k = 31$ in a genome-skim, we used

```
jellyfish count -m 31 -s 100M -C -o COUNT_FILE FASTQ_FILE
```

and to get the histogram of k-mer counts

```
jellyfish histo COUNT_FILE
```

Computing Jaccard index and estimating distance using Mash

We first *sketch* input genome-skims or assemblies with k-mer length $k = 31$ and sketch size $s = 10^7$. For genome-skims (in FASTQ format) when no k-mer filtering is applied, we run

```
mash sketch -r -k 31 -s 10000000 -o SKETCH_FILE FASTQ_FILE
```

To sketch genome-skims while filtering k-mers with less than C copies, we use

```
mash sketch -m C -k 31 -s 10000000 -o SKETCH_FILE FASTQ_FILE
```

For genome assemblies (in FASTA format), we used

```
mash sketch -k 31 -s 10000000 -o SKETCH_FILE FASTA_FILE
```

Then, the Jaccard index and Mash distance between sketches is computed by running

```
mash dist SKETCH_FILE_1 SKETCH_FILE_2
```

Estimating distances using AAF

To count the k-mers ($k = 31$) in a dataset of genome-skims using 24 cores and 120GB memory, we first ran

```
python PATH_to_FILE/aaf_phylokmer.py -k 31 -t 24 -o KMER_COUNT_FILE \
        -d INPUT_DIR -G 120
```

Next, to get the (uncorrected) distances and phylogeny, we used

```
python PATH_to_FILE/aaf_distance.py -i KMER_COUNT_FILE -t 24 -G 120 \
        -o OUTPUT_FILE_PREFIX -f KMER_DIVERSITY_FILE
```

where KMER_DIVERSITY_FILE is an output of previous command. Finally, to correct tip branches of phylogeny tree for low coverage and sequencing error, we used

```
python PATH_to_FILE/aaf_tip.py -i TREE_FILE -k 31 \
        --tip TIP_INFO_FILE -f KMER_DIVERSITY_FILE
```

where we had to provide TIP_INFO_FILE containing estimates of coverage and sequencing error. To estimate coverage, we followed the procedure suggested in AAF user manual. We first used JellyFish to find the k-mer counts M_i's as described before. They suggest when there is a clear peak in the k-mer frequency distribution, estimate k-mer coverage λ to be the maximum bin. As they do not suggest a specific rule for that, we first find $j = \operatorname{argmax}_{i>1} M_i$, excluding the count of the first bin M_1, which is always large because of erroneous k-mers due to sequencing error. If $j > 2$, it means that we can see a peak in k-mers distribution at j, so we use $\lambda = j$. Otherwise, if $j = 2$, we follow their suggested formula $\lambda = \frac{\sum iM_i}{\sum M_i}$ for the case of low coverage or high sequencing error that there is no clear peak in the k-mer frequency distribution. We should also mention that no k-mer filtering used for AAF, as the coverage was heterogeneous over genome-skims. In fact, in AAF the filtering is applied to all genome-skims if used, and so they suggest to not apply filtering when there is any taxon with low coverage ($c < 5$) within the dataset.

Preprocessing raw reads using fastp

We used the following command to filter low-quality reads and trim the adapter sequences

```
fastp -t 1 -i INPUT_READS_R1 -I INPUT_READS_R2 \
      -o OUTPUT_READS_R1 -O OUTPUT_READS_R2
```

Contamination removal

To remove bacterial and mitochondrial sequences, we first created a BLAST database from the assemblies of contaminant genomes by running

```
makeblastdb -in CONTAMINANTS_FASTA_FILE -dbtype nucl -out BLAST_DB
```

and then searched the reads against these genomes using Megablast

```
blastn -db BLAST_DB -query READS_FILE -outfmt 6 -out MEGABLAST_OUTPUT
```

We also used Bowtie2 to find the reads aligned to the human reference genome

```
bowtie2 -x HUMAN_REFERENCE -U READS_FILE \
        -S BOWTIE_OUTPUT --very-sensitive-local
```

We then removed any read found in `MEGABLAST_OUTPUT` or `BOWTIE_OUTPUT`.

Appendix D: Supplementary figures and tables

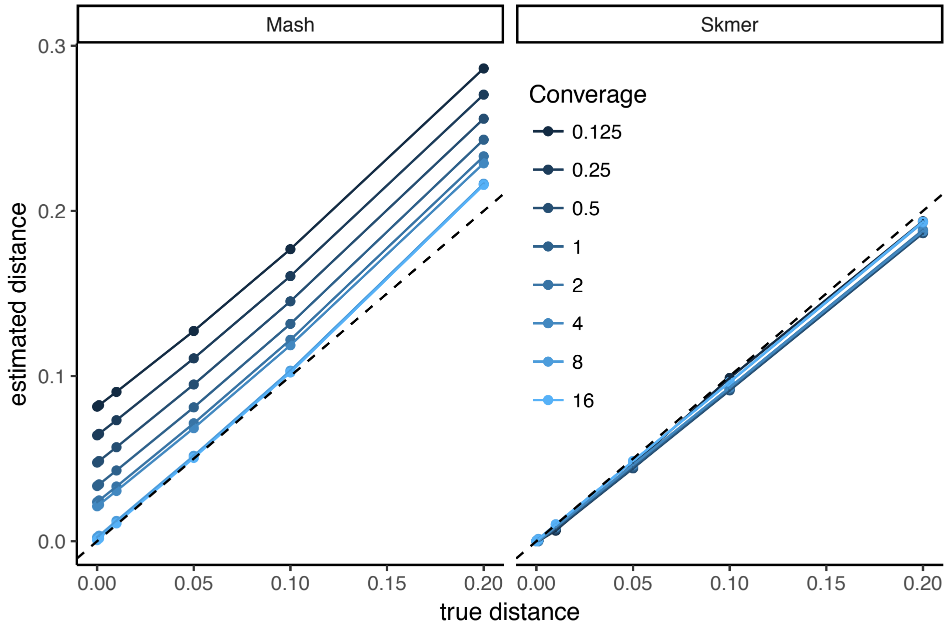

Figure S1: **Comparing the accuracy of Mash and Skmer on simulated genomes.** Genome-skims are simulated using ART with read length $\ell = 100$. Substitutions applied to the assembly of *C. vestalis* at six different rates (x-axis), and genome-skims simulated at varying coverage range from $\frac{1}{8}$X to 16X (colors). The estimated distance (y-axis) by Mash (left) and Skmer (right) is plotted versus the real distances (x-axis). The mean (dots) distances are shown as dots (10 repeats) but standard errors are too small to see. The unit line is shown as a dashed line.

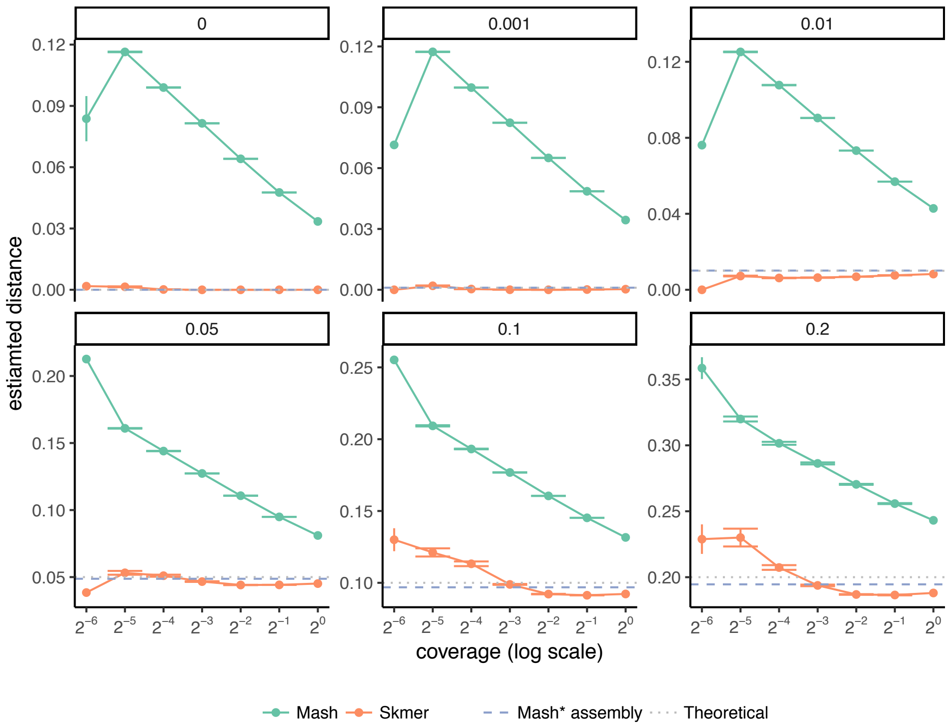

Figure S2: **Comparing distances estimated by Mash and Skmer for simulated data at very low coverages.** Skims of *C. vestalis* v.s. genomes simulated to be at different distances from *C. vestalis*, with varying coverage. The mean and standard error of distances are shown over 10 repeats of the experiment. The coverage ranges from $\frac{1}{64}$X to 1X.

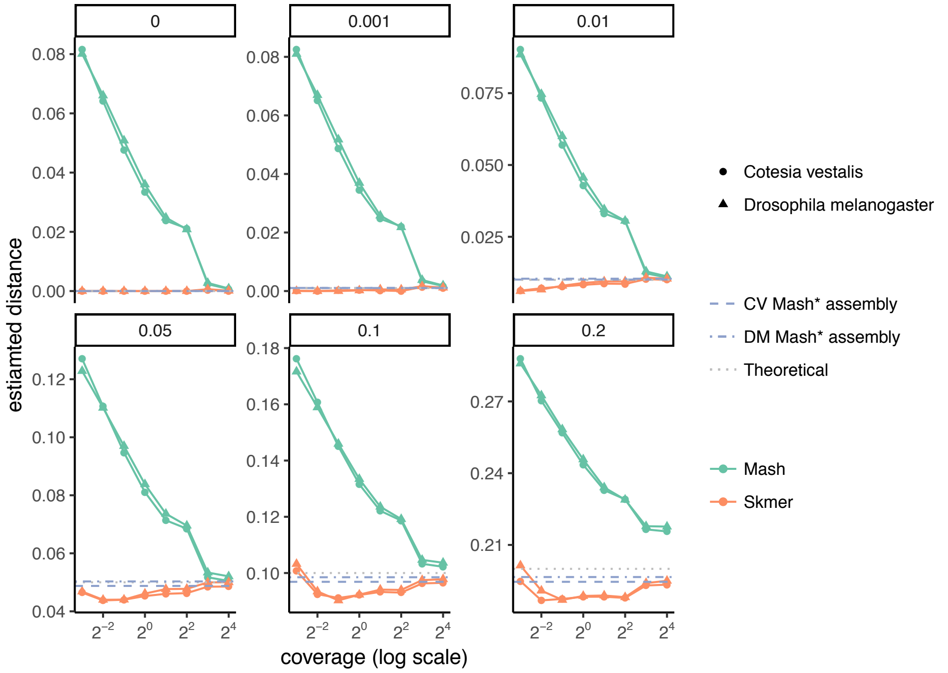

Figure S3: **Comparing distances estimated for genome-skims of two different species.** Genomes simulated at different distances from the genomes of C. vestalis and D. melanogaster and subsampled at a range of coverage from $\frac{1}{8}$X to 16X.

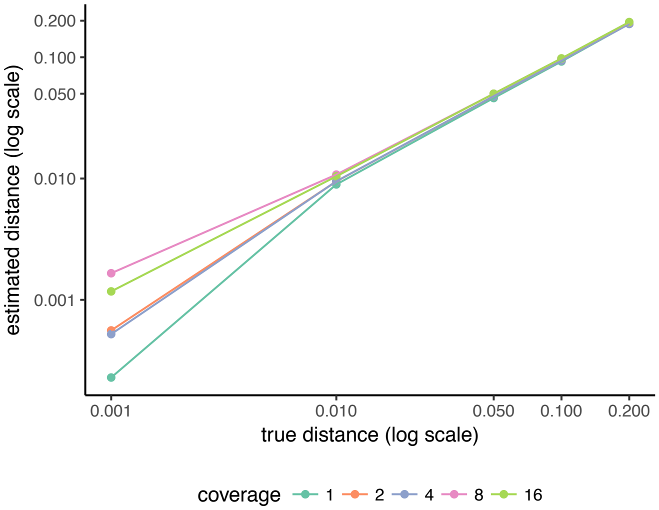

(a)

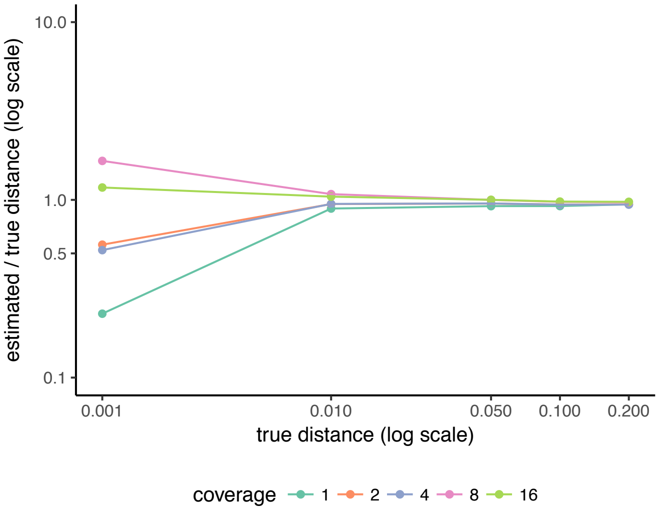

(b)

Figure S4: **The resolution of Skmer at different genomic distances.** Skims of *D. melanogaster* v.s. genomes simulated to be at different distances from *D. melanogaster*, with varying coverage. (a) Estimated distance versus the true distance. (b) The ratio of estimated distance to the true distance.

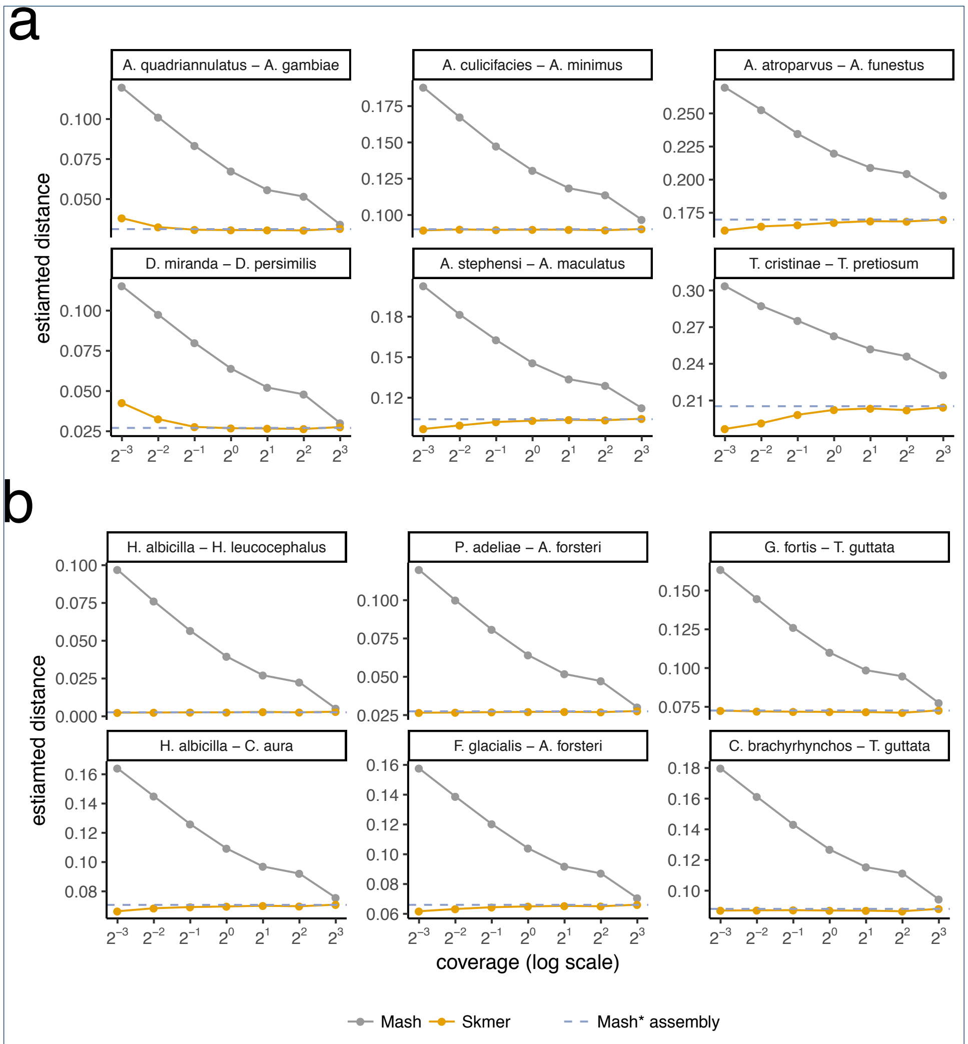

Figure S5: **Comparing the accuracy of Mash and Skmer on pairs of insects and birds genomes.** Genome-skims simulated at coverage $\frac{1}{8}$X to 8X. On each subplot, the estimated distance (y-axis) is plotted versus the coverage (x-axis) for a pair of species. Dashed line shows Mash* run on assemblies, which is taken as the true distance. Skmer estimates (light-colored curves) are very close to the true distance while Mash (gray curves) largely overestimates at lower coverages. (**a**) Six pairs of insects. (**b**) Six pairs of birds.

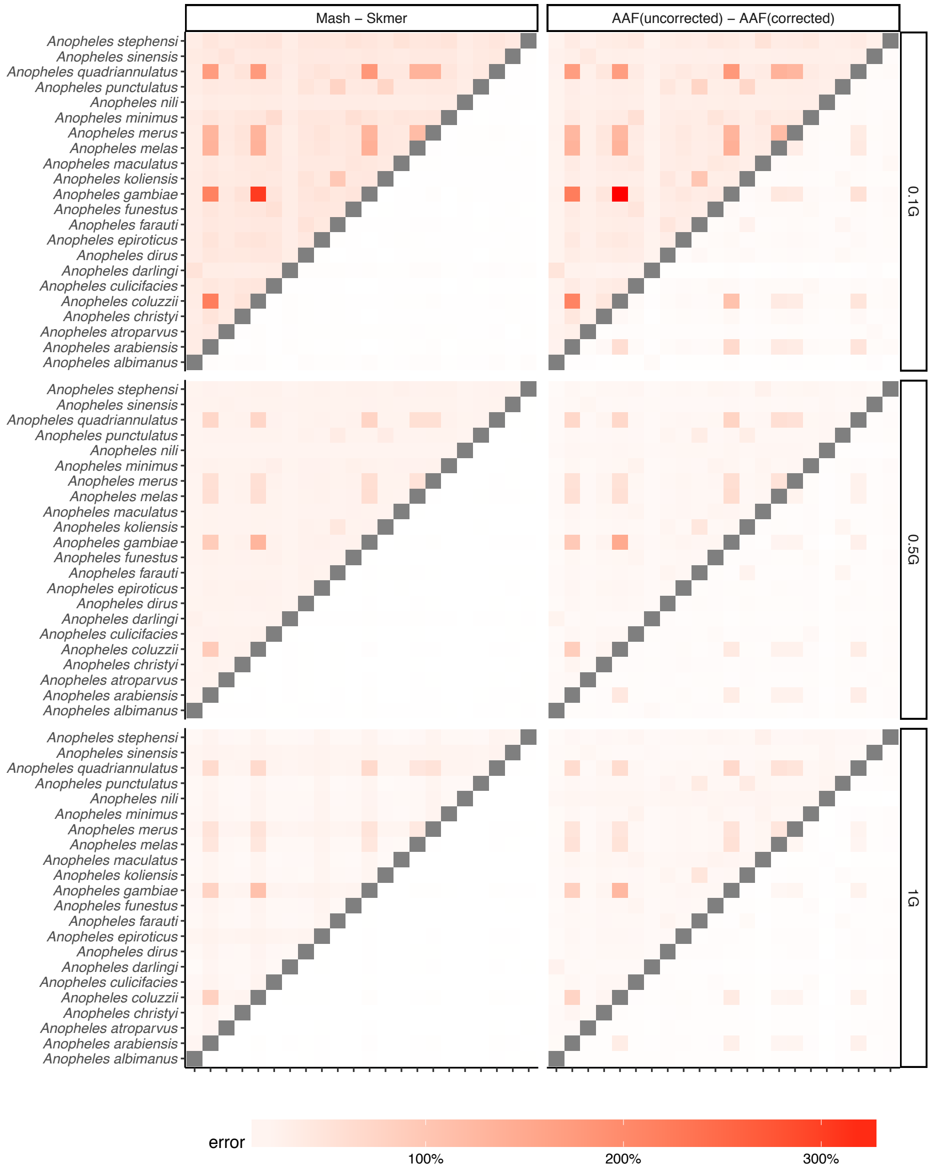

Figure S6: **Comparing the error of Mash, Skmer, and AAF in distance estimation with fixed amount of sequence from each species.** The dataset of 22 Anopheles genomes, subsampled with 0.1Gb, 0.5Gb, and 1Gb sequence.

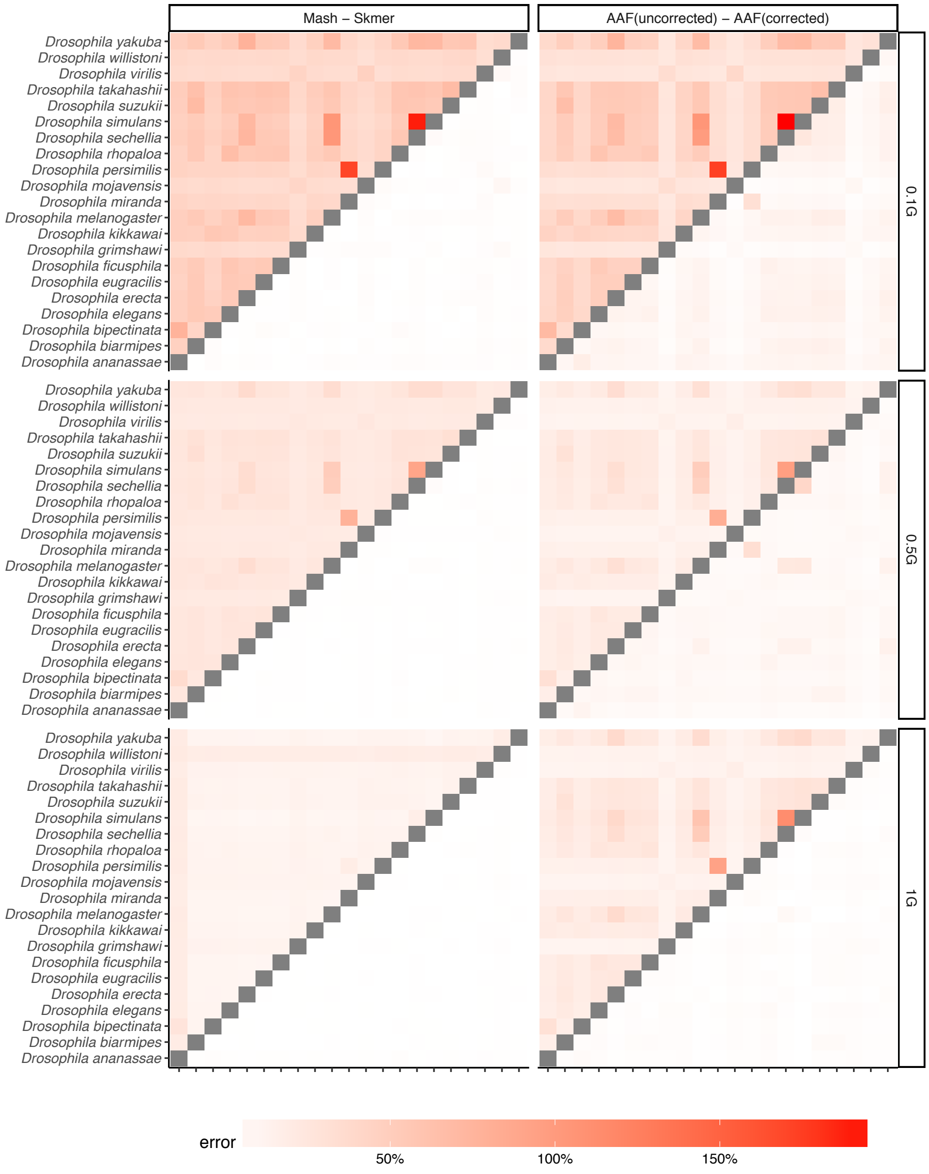

Figure S7: **Comparing the error of Mash, Skmer, and AAF in distance estimation with fixed amount of sequence from each species.** The dataset of 21 Drosophila genomes, subsampled with 0.1Gb, 0.5Gb, and 1Gb sequence.

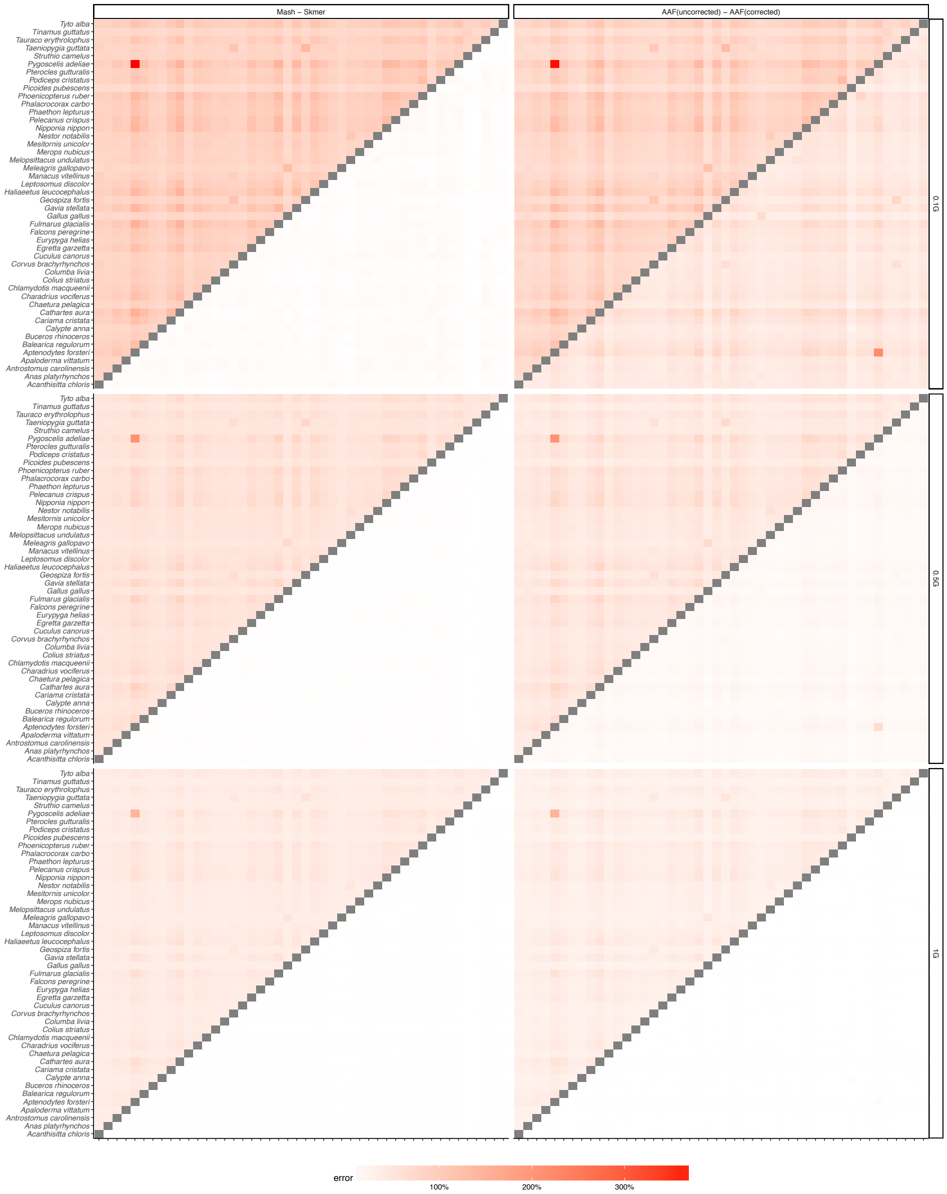

Figure S8: **Comparing the error of Mash, Skmer, and AAF in distance estimation with fixed amount of sequence from each species.** The dataset of 47 avian genomes, subsampled with 0.1Gb, 0.5Gb, and 1Gb sequence.

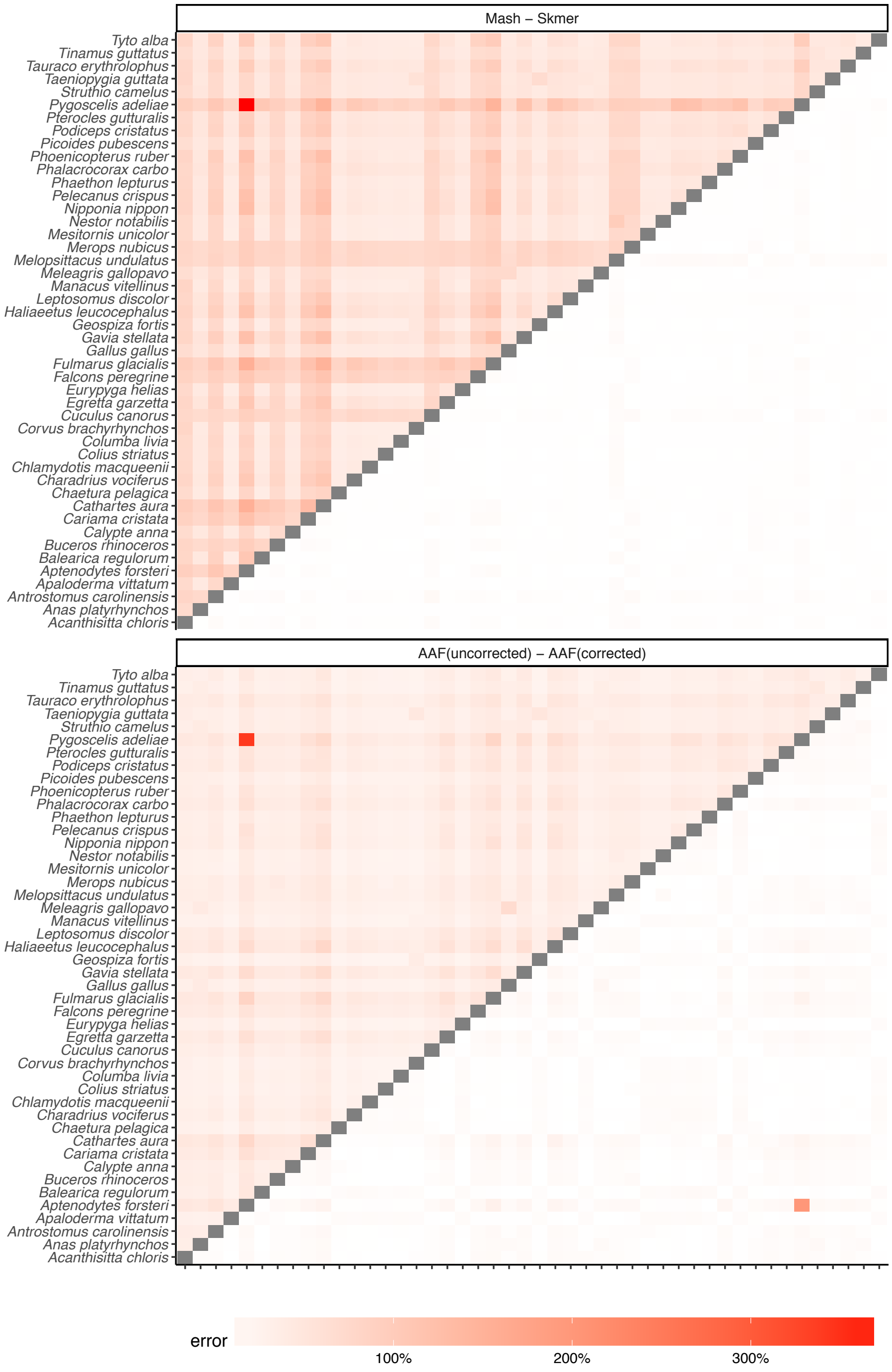

Figure S9: **Comparing the error of Mash, Skmer, and AAF on the Avian dataset with mixed coverage.** Species have random amount of sequence chosen uniformly among 0.1Gb, 0.5Gb, and 1Gb. Similar to (Fig. 5), we have excluded one of the eagles (*H. albicilla*). The error of Mash, AAF, and Skmer in estimating the distance between the two eagles are 2193%, 884%, and 4.2%, respectively (both of the eagles are subsampled at 0.5Gb here).

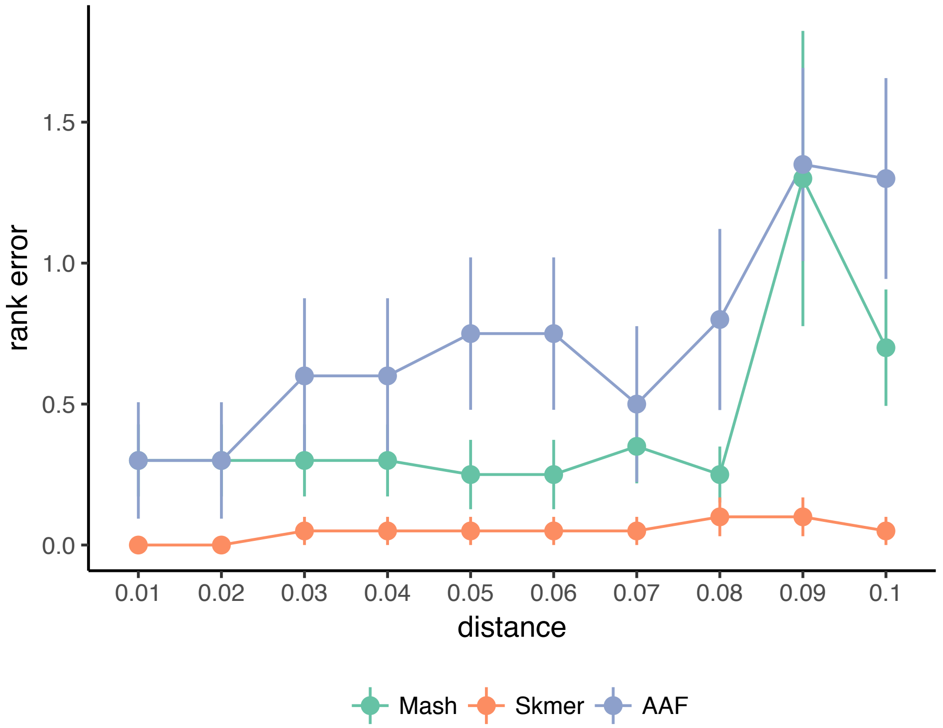

Figure S10: **The mean rank error of the best remaining match in leave-out experiments on the *Drosophila* dataset.** *Drosophila willistoni* has been excluded.

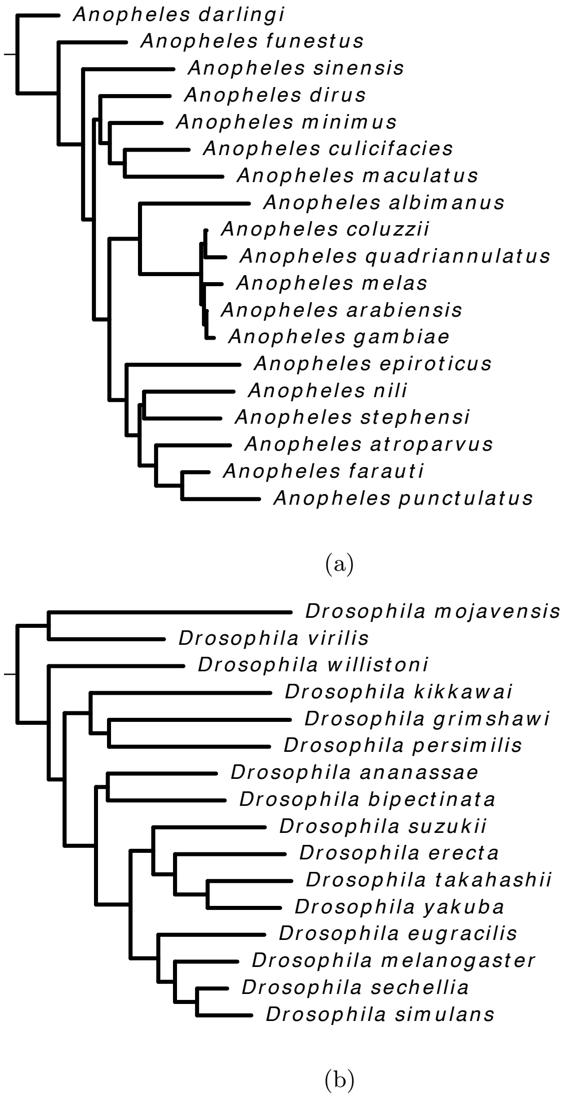

Figure S11: **Maximum-likelihood trees inferred from COI barcodes** (a) *Anopheles* tree. (b) *Drosophila* tree.

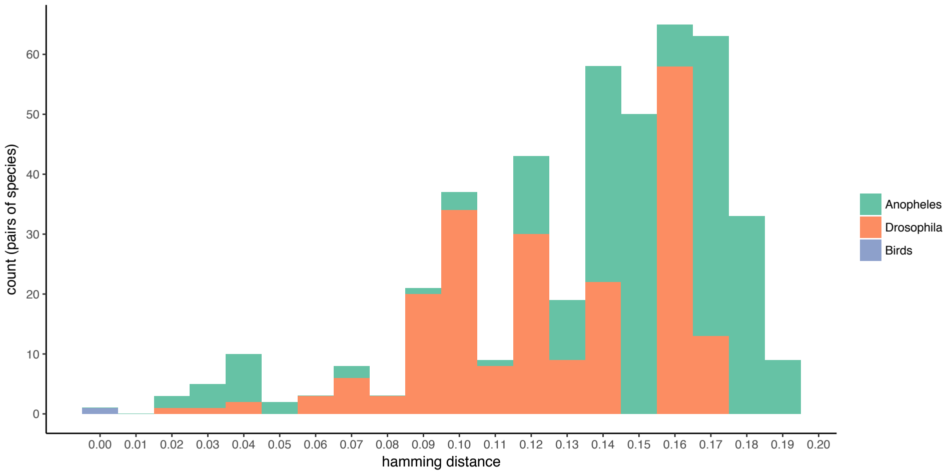

Figure S12: **The histogram of genomic distances between species from the same genus among the Anopheles, Drosophila, and birds datasets.** Distances computed based on full assemblies. The only species from the same genus with hamming distance less than 0.01 were the two eagle species (*H. albicilla* and *H. leucocephalus*).

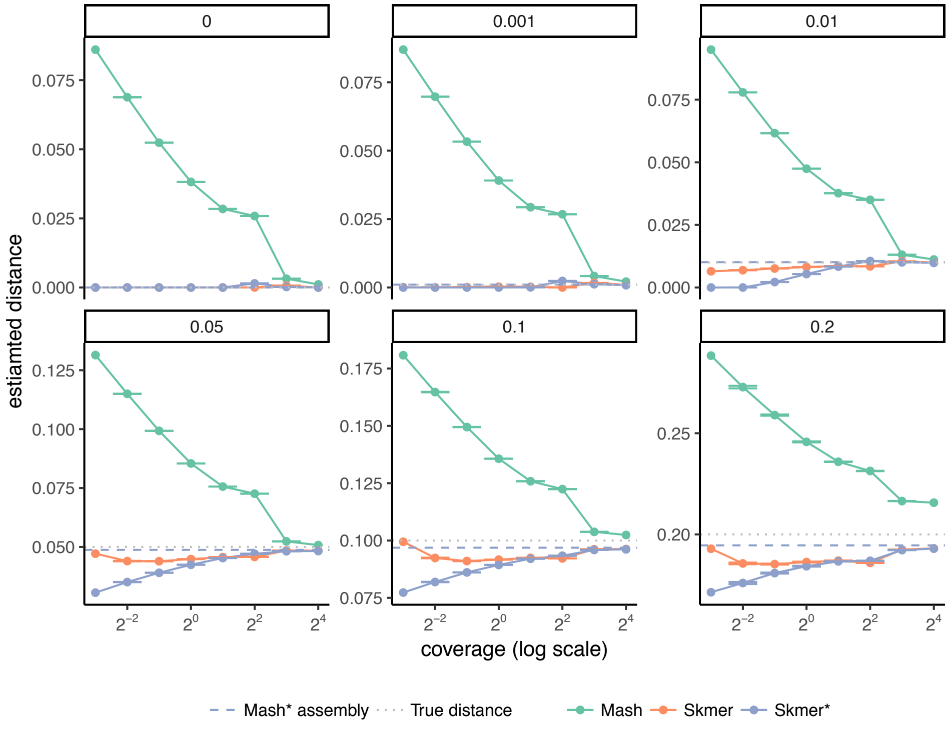

Figure S13: **The performance of Skmer coverage estimation.** Comparing distances estimated by Mash, Skmer with estimated coverages, and Skmer with true coverages (Skmer*), on genome-skims of *C. vestalis* and genomes simulated at different distances from it.

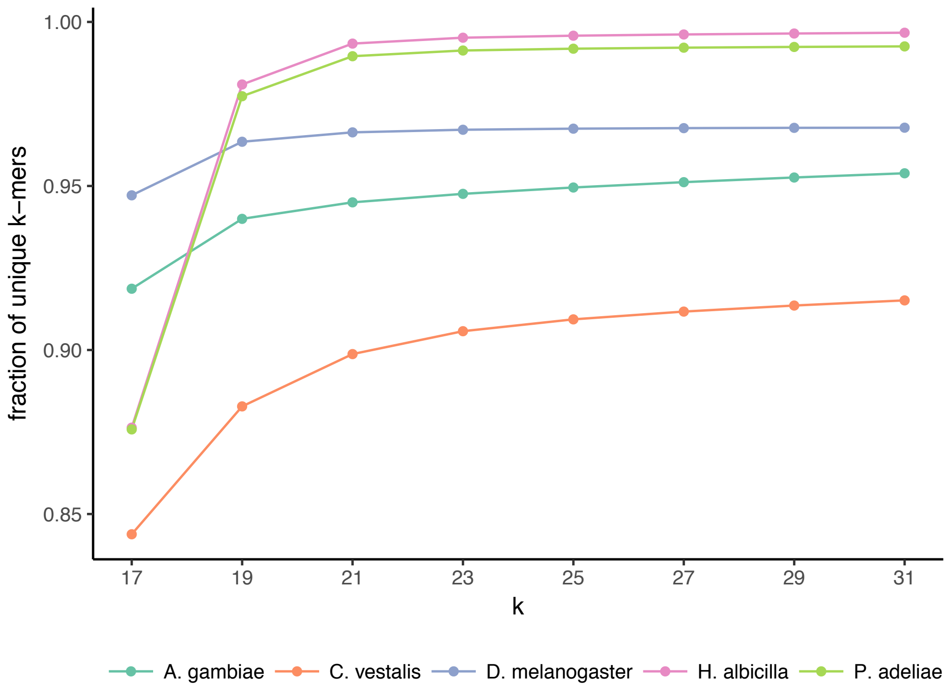

Figure S14: **The fraction of unique k-mers in selected species of insects and birds.**

Table S1: GenBank accession numbers of microbial species used in contamination removal.

Species	GenBank assembly accession
Pasteurella langaaensis	GCA_003096995.1
Providencia stuartii	GCA_001558855.2
Serratia marcescens	GCA_000783915.2
Shigella flexneri	GCA_000006925.2
Commensalibacter intestini	GCA_002153535.1
Acetobacter malorum	GCA_002153605.1
Acetobacter pomorum	GCA_002456135.1
Lactobacillus plantarum	GCA_000203855.3
Lactobacillus brevis	GCA_003184305.1
Enterococcus faecalis	GCA_002208945.2
Vagococcus teuberi	GCA_001870205.1
Wolbachia	GCA_000022285.1

Table S2: GenBank accession numbers and URLs for Anopheles genomes

Species	GenBank assembly accession	URL
Anopheles albimanus	GCA_000349125.1	http://www.insect-genome.com/data/genome_download/Anopheles_albimanus/Anopheles_albimanus_genomic.fasta.gz
Anopheles arabiensis	GCA_000349185.1	http://www.insect-genome.com/data/genome_download/Anopheles_arabiensis/Anopheles_arabiensis_genomic.fasta.gz
Anopheles atroparvus	GCA_000473505.1	http://www.insect-genome.com/data/genome_download/Anopheles_atroparvus/Anopheles_atroparvus_genomic.fasta.gz
Anopheles christyi	GCA_000349165.1	http://www.insect-genome.com/data/genome_download/Anopheles_christyi/Anopheles_christyi_genomic.fasta.gz
Anopheles coluzzii	-	http://www.insect-genome.com/data/genome_download/Anopheles_coluzzii/Anopheles_coluzzii_genomic.fasta.gz
Anopheles culicifacies	GCA_000473375.1	http://www.insect-genome.com/data/genome_download/Anopheles_culicifacies/Anopheles_culicifacies_genomic.fasta.gz
Anopheles darlingi	GCA_000211455.3	http://www.insect-genome.com/data/genome_download/Anopheles_darlingi/Anopheles_darlingi_genomic.fasta.gz
Anopheles dirus	GCA_000349145.1	http://www.insect-genome.com/data/genome_download/Anopheles_dirus/Anopheles_dirus_genomic.fasta.gz
Anopheles epiroticus	GCA_000349105.1	http://www.insect-genome.com/data/genome_download/Anopheles_epiroticus/Anopheles_epiroticus_genomic.fasta.gz
Anopheles farauti	GCA_000956265.1	http://www.insect-genome.com/data/genome_download/Anopheles_farauti/Anopheles_farauti_genomic.fasta.gz
Anopheles funestus	GCA_000349085.1	http://www.insect-genome.com/data/genome_download/Anopheles_funestus/Anopheles_funestus_genomic.fasta.gz
Anopheles gambiae	GCA_000150785.1	http://www.insect-genome.com/data/genome_download/Anopheles_gambiae/Anopheles_gambiae_genomic.fasta.gz
Anopheles koliensis	GCA_000956275.1	http://www.insect-genome.com/data/genome_download/Anopheles_koliensis/Anopheles_koliensis_genomic.fasta.gz
Anopheles maculatus	GCA_000473185.1	http://www.insect-genome.com/data/genome_download/Anopheles_maculatus/Anopheles_maculatus_genomic.fasta.gz
Anopheles melas	GCA_000473525.2	http://www.insect-genome.com/data/genome_download/Anopheles_melas/Anopheles_melas_genomic.fasta.gz
Anopheles merus	GCA_000473845.2	http://www.insect-genome.com/data/genome_download/Anopheles_merus/Anopheles_merus_genomic.fasta.gz
Anopheles minimus	GCA_000349025.1	http://www.insect-genome.com/data/genome_download/Anopheles_minimus/Anopheles_minimus_genomic.fasta.gz
Anopheles nili	GCA_000439205.1	http://www.insect-genome.com/data/genome_download/Anopheles_nili/Anopheles_nili_genomic.fasta.gz
Anopheles punctulatus	GCA_000956255.1	http://www.insect-genome.com/data/genome_download/Anopheles_punctulatus/Anopheles_punctulatus_genomic.fasta.gz
Anopheles quadriannulatus	GCA_000349065.1	http://www.insect-genome.com/data/genome_download/Anopheles_quadriannulatus/Anopheles_quadriannulatus_genomic.fasta.gz
Anopheles sinensis	GCA_000441895.2	http://www.insect-genome.com/data/genome_download/Anopheles_sinensis/Anopheles_sinensis_genomic.fasta.gz
Anopheles stephensi	GCA_000300775.2	http://www.insect-genome.com/data/genome_download/Anopheles_stephensi/Anopheles_stephensi_genomic.fasta.gz

Table S3: GenBank accession numbers and URLs for Drosophila genomes

Species	GenBank assembly accession	URL
Drosophila ananassae	GCA_000005115.1	http://www.insect-genome.com/data/genome_download/Drosophila_ananassae/Drosophila_ananassae_genomic.fasta.gz
Drosophila biarmipes	GCA_000233415.2	http://www.insect-genome.com/data/genome_download/Drosophila_biarmipes/Drosophila_biarmipes_genomic.fasta.gz
Drosophila bipectinata	GCA_000236285.2	http://www.insect-genome.com/data/genome_download/Drosophila_bipectinata/Drosophila_bipectinata_genomic.fasta.gz
Drosophila elegans	GCA_000224195.2	http://www.insect-genome.com/data/genome_download/Drosophila_elegans/Drosophila_elegans_genomic.fasta.gz
Drosophila erecta	GCA_000005135.1	http://www.insect-genome.com/data/genome_download/Drosophila_erecta/Drosophila_erecta_genomic.fasta.gz
Drosophila eugracilis	GCA_000236325.2	http://www.insect-genome.com/data/genome_download/Drosophila_eugracilis/Drosophila_eugracilis_genomic.fasta.gz
Drosophila ficusphila	GCA_000220665.2	http://www.insect-genome.com/data/genome_download/Drosophila_ficusphila/Drosophila_ficusphila_genomic.fasta.gz
Drosophila grimshawi	GCA_000005155.1	http://www.insect-genome.com/data/genome_download/Drosophila_grimshawi/Drosophila_grimshawi_genomic.fasta.gz
Drosophila kikkawai	GCA_000224215.2	http://www.insect-genome.com/data/genome_download/Drosophila_kikkawai/Drosophila_kikkawai_genomic.fasta.gz
Drosophila melanogaster	GCA_000778455.1	http://www.insect-genome.com/data/genome_download/Drosophila_melanogaster/Drosophila_melanogaster_genomic.fasta.gz
Drosophila miranda	GCA_000269505.2	http://www.insect-genome.com/data/genome_download/Drosophila_miranda/Drosophila_miranda_genomic.fasta.gz
Drosophila mojavensis	GCA_000005175.1	http://www.insect-genome.com/data/genome_download/Drosophila_mojavensis/Drosophila_mojavensis_genomic.fasta.gz
Drosophila persimilis	GCA_000005195.1	http://www.insect-genome.com/data/genome_download/Drosophila_persimilis/Drosophila_persimilis_genomic.fasta.gz
Drosophila rhopaloa	GCA_000236305.2	http://www.insect-genome.com/data/genome_download/Drosophila_rhopaloa/Drosophila_rhopaloa_genomic.fasta.gz
Drosophila sechellia	GCA_000005215.1	http://www.insect-genome.com/data/genome_download/Drosophila_sechellia/Drosophila_sechellia_genomic.fasta.gz
Drosophila simulans	GCA_000259055.1	http://www.insect-genome.com/data/genome_download/Drosophila_simulans/Drosophila_simulans_genomic.fasta.gz
Drosophila suzukii	GCA_000472105.1	http://www.insect-genome.com/data/genome_download/Drosophila_suzukii/Drosophila_suzukii_genomic.fasta.gz
Drosophila takahashii	GCA_000224235.2	http://www.insect-genome.com/data/genome_download/Drosophila_takahashii/Drosophila_takahashii_genomic.fasta.gz
Drosophila virilis	GCA_000005245.1	http://www.insect-genome.com/data/genome_download/Drosophila_virilis/Drosophila_virilis_genomic.fasta.gz
Drosophila willistoni	GCA_000005925.1	http://www.insect-genome.com/data/genome_download/Drosophila_willistoni/Drosophila_willistoni_genomic.fasta.gz
Drosophila yakuba	GCA_000005975.1	http://www.insect-genome.com/data/genome_download/Drosophila_yakuba/Drosophila_yakuba_genomic.fasta.gz

Table S4: GenBank accession numbers and URLs for avian genomes

Species	GenBank assembly accession	URL
Acanthisitta chloris	GCA_000695815.1	http://dx.doi.org/10.5524/101015
Anas platyrhynchos	GCA_000355885.1	http://dx.doi.org/10.5524/101001
Antrostomus carolinensis	GCA_000700745.1	http://dx.doi.org/10.5524/101019
Apaloderma vittatum	GCA_000703405.1	http://dx.doi.org/10.5524/101016
Aptenodytes forsteri	GCA_000699145.1	http://dx.doi.org/10.5524/100005
Balearica regulorum	GCA_000709895.1	http://dx.doi.org/10.5524/101017
Buceros rhinoceros	GCA_000710305.1	http://dx.doi.org/10.5524/101018
Calypte anna	GCA_000699085.1	http://dx.doi.org/10.5524/101004
Cariama cristata	GCA_000690535.1	http://dx.doi.org/10.5524/101020
Cathartes aura	GCA_000699945.1	http://dx.doi.org/10.5524/101021
Chaetura pelagica	GCA_000747805.1	http://dx.doi.org/10.5524/101005
Charadrius vociferus	GCA_000708025.2	http://dx.doi.org/10.5524/101007
Chlamydotis macqueenii	GCA_000695195.1	http://dx.doi.org/10.5524/101022
Colius striatus	GCA_000690715.1	http://dx.doi.org/10.5524/101023
Columba livia	GCA_000337935.1	http://dx.doi.org/10.5524/100007
Corvus brachyrhynchos	GCA_000691975.1	http://dx.doi.org/10.5524/101008
Cuculus canorus	GCA_000709325.1	http://dx.doi.org/10.5524/101009
Egretta garzetta	GCA_000687185.1	http://dx.doi.org/10.5524/101002
Eurypyga helias	GCA_000690775.1	http://dx.doi.org/10.5524/101024
Falcons peregrine	GCA_000337955.1	http://dx.doi.org/10.5524/101006
Fulmarus glacialis	GCA_000690835.1	http://dx.doi.org/10.5524/101025
Gallus gallus	GCA_000002315.3	ftp://climb.genomics.cn/pub/10.5524/100001_101000/101000/chicken/
Gavia stellata	GCA_000690875.1	http://dx.doi.org/10.5524/101026
Geospiza fortis	GCA_000277835.1	http://dx.doi.org/10.5524/100040
Haliaeetus albicilla	GCA_000691405.1	http://dx.doi.org/10.5524/101027
Haliaeetus leucocephalus	GCA_000737465.1	http://dx.doi.org/10.5524/101040
Leptosomus discolor	GCA_000691785.1	http://dx.doi.org/10.5524/101028
Manacus vitellinus	GCA_000692015.2	http://dx.doi.org/10.5524/101010
Meleagris gallopavo	GCA_000146605.3	ftp://climb.genomics.cn/pub/10.5524/100001_101000/101000/turkey/
Melopsittacus undulatus	GCA_000238935.1	http://dx.doi.org/10.5524/100059
Merops nubicus	GCA_000691845.1	http://dx.doi.org/10.5524/101029
Mesitornis unicolor	GCA_000695765.1	http://dx.doi.org/10.5524/101030
Nestor notabilis	GCA_000696875.1	http://dx.doi.org/10.5524/101031
Nipponia nippon	GCA_000708225.1	http://dx.doi.org/10.5524/101003
Pelecanus crispus	GCA_000687375.1	http://dx.doi.org/10.5524/101032
Phaethon lepturus	GCA_000687285.1	http://dx.doi.org/10.5524/101033
Phalacrocorax carbo	GCA_000708925.1	http://dx.doi.org/10.5524/101034
Phoenicopterus ruber	GCA_000687265.1	http://dx.doi.org/10.5524/101035
Picoides pubescens	GCA_000699005.1	http://dx.doi.org/10.5524/101012
Podiceps cristatus	GCA_000699545.1	http://dx.doi.org/10.5524/101036
Pterocles gutturalis	GCA_000699245.1	http://dx.doi.org/10.5524/101037
Pygoscelis adeliae	GCA_000699105.1	http://dx.doi.org/10.5524/100006
Struthio camelus	GCA_000698965.1	http://dx.doi.org/10.5524/101013
Taeniopygia guttata	GCA_000151805.2	ftp://climb.genomics.cn/pub/10.5524/100001_101000/101000/zebrafinch/
Tauraco erythrolophus	GCA_000709365.1	http://dx.doi.org/10.5524/101038
Tinamus guttatus	GCA_000705375.2	http://dx.doi.org/10.5524/101014
Tyto alba	GCA_000687205.1	http://dx.doi.org/10.5524/101039

Table S5: **The coverage of genomes over three datasets.** Each genome is skimmed with 100Mb sequence.

Dataset	Min	Mean	Max
Drosophila	0.45X	0.60X	0.79X
Anopheles	0.37X	0.57X	1.02X
Birds	0.082X	0.090X	0.107X

Table S6: **Comparing the average error of Mash, Skmer, and AAF over three datasets.** Fixed sequencing effort from each species.

Dataset	Sequencing effort	Mash	Skmer	AAF (uncorrected)	AAF (corrected)
	0.1Gb	48.02% (1.54%)	2.02% (0.05%)	40.22% (1.67%)	9.62% (0.52%)
Anopheles	0.5Gb	24.89% (0.59%)	0.75% (0.02%)	17.60% (0.70%)	7.35% (0.26%)
	1Gb	18.43% (0.54%)	0.55% (0.02%)	16.94% (0.61%)	4.74% (0.22%)
	0.1Gb	47.98% (0.82%)	1.65% (0.06%)	40.67% (0.94%)	9.00% (0.20%)
Drosophila	0.5Gb	25.25% (0.34%)	0.72% (0.03%)	18.63% (0.45%)	7.00% (0.19%)
	1Gb	13.00% (0.16%)	0.50% (0.02%)	19.69% (0.52%)	2.18% (0.06%)
	0.1Gb	95.57% (2.54%)	5.72% (0.06%)	86.45% (3.18%)	49.48% (1.94%)
Birds	0.5Gb	56.61% (1.40%)	2.14% (0.02%)	49.13% (1.75%)	13.73% (0.56%)
	1Gb	41.25% (0.97%)	1.32% (0.01%)	34.33% (1.22%)	1.05% (0.09%)

* The standard error of the mean is provided in parentheses.
